# Advancing clinical insight into creatine transporter deficiency: long term outcome and new observations from the Italian cohort

**DOI:** 10.1186/s13023-026-04289-3

**Published:** 2026-03-03

**Authors:** Maria Grazia Alessandrì, Chiara Bosetti, Elena Scaffei, Claudia Casalini, Simona Balestrini, Luciana Tramacere, Pierfrancesco Ambrosi, Michela Tosetti, Renzo Guerrini, Roberta Battini

**Affiliations:** 1 IRCCS Stella Maris Foundation, viale del Tirreno 331, Calambrone, Pisa 56128 Italy; 2 Department of Clinical and Experimental Medicine, University of Pisa, Via Roma 55, Pisa, 56126 Italy; 3 Neuroscience and Medical Genetics Department, Meyer Children’s Hospital IRCCS, Viale Pieraccini 24, Firenze, 50139 Italy; 4 NEUROFARBA Department, University of Florence, Viale Pieraccini 6, Firenze, 50139 Italy; 5 Department of Medicine, Unit of Neurology of Florence, “San Giovanni di Dio” Hospital, Via di Torregalli 3, Firenze, 50143 Italy

**Keywords:** Creatine transporter deficiency (CTD), SLC6A8 gene, Cerebral creatine deficiency disorders, X-linked intellectual disabilities, Arginine treatment, Magnetic resonance spectroscopy

## Abstract

**Background:**

Creatine Transporter Deficiency (CTD) is a rare X-linked disorder caused by pathogenic or likely pathogenic variants in the *SLC6A8* gene, leading to a deficiency of cerebral Creatine. Clinically, CTD manifests as a complex neurodevelopmental disorder and is associated with Intellectual Disability (ID), language and socio-communicative impairments, behavioral challenges and, often, epilepsy.

**Methods:**

This study was conducted in two phases: (i) An eSurvey was distributed among major Italian clinics specializing in rare neurometabolic diseases to create a national census of CTD patients, with extensive clinical data; (ii) A retrospective observational study was performed on patients who had undergone regular long-term follow-up using a consistent neuropsychological assessment and treatment protocol.

**Results:**

We identified 18 CTD male patients, aged 18 months to 32 years at diagnosis. Within this cohort, 3 to 13 years follow-up clinical information was collected for 10 patients. A specific clinical protocol was applied to this subgroup, where a biochemical or ^1^H-MRS diagnosis was confirmed in 8 patients (urine Creatine/Creatinine ratio > 1), while in the remaining 2 patients, genetic testing was diagnostic and neurochemical tests followed. ^1^H-MRS consistently showed a decreased Creatine peak in all patients. All 10 patients exhibited ID with significant speech disorder, and 6 had epilepsy. The treatment protocol for this cohort involved oral Arginine supplementation.

**Conclusions:**

Diagnosing CTD remains clinically challenging due to the often negative results from a first tier clinical diagnostic test for intellectual disability (ID) (i.e. family history, cytogenetic testing and Fragile X) and neuroimaging without spectroscopy. CTD should be carefully considered when investigating severe ID with autistic-like traits and significant speech impairment, with or without epilepsy. The Urine Creatine/Creatinine ratio assay is a quick initial diagnostic step, which can be corroborated by a brain MRI/^1^H-MRS and molecular genetics. Even considering previouis literature findings, it is not yet possible, to demonstrate definite genotype-phenotype correlations, although a milder functional impairment has been suggested for missense *SLC6A8* pathogenic or likely pathogenic variants. Treatment strategies supplementation with Creatine and its precursors have provided heterogeneous and inconsistent results. Recently proposed innovative therapeutic strategies such as lipophilic Creatine analogs and betaine supplementation in animal models need validation in human disease.

**Supplementary Information:**

The online version contains supplementary material available at 10.1186/s13023-026-04289-3.

## Background

Creatine is a crucial guanidinium compound synthesized from arginine, glycine, and methionine, essential for cellular energy homeostasis [1]. Approximately 50% of the body’s Creatine is obtained through diet, primarily from meat and fish, while the other 50% is endogenously synthesized [1]. The synthesis pathway involves two steps: the first, catalyzed by Arginine: glycine amidinotransferase (AGAT), produces guanidinoacetate (GAA), and is the rate-limiting step of the reaction. The second step is catalyzed by Guanidinoacetate methyltransferase (GAMT), which synthesizes creatine [1–4]. The synthesis primarily occurs in the liver, kidney, and pancreas [1]. From these sites, Creatine is distributed to high-energy demand tissues like skeletal muscle and the brain. Since the brain constitutes only 2% of body mass but consumes 20% of total energy, it requires a significant and rapid Adenosine Triphosphate (ATP) turnover, largely supported by the creatine/phosphocreatine system [1–3, 5, 6]. Creatine must cross the blood-brain barrier (BBB) to reach neural cells, a process mediated by the specific creatine transporter, CRT (SLC6A8). Therefore, CRT plays a pivotal role in supplying the brain with peripherally synthesized creatine, making it indispensable for proper neuronal energy metabolism and function [2, 7]. Although some endogenous creatine synthesis occurs within the brain, its reliance on the transporter for peripheral uptake highlights the critical importance of CRT [5, 8].

Cerebral Creatine Deficiency Syndromes (CCDS) comprise a group of rare inborn errors of Creatine metabolism, including AGAT deficiency (OMIM 602360), GAMT deficiency (OMIM 601240) and Creatine Transporter Deficiency (CTD) (OMIM 300352). Among these, CTD is an X-linked inherited metabolic disorder caused by pathogenic or likely pathogenic variants in the *SLC6A8* gene on Xq28, which encodes the Creatine transporter. Clinically, CTD manifests as a severe neurodevelopmental disorder with early-onset global developmental delay and cognitive dysfunction, speech and language impairment, epilepsy and autistic-like behaviors [9]. Cardiac [10] and gastrointestinal manifestations [11], have been also reported, particularly in adults.

Hemyzigous females with Creatine Transporter Deficiency (CTD) exhibit a wide phenotypic spectrum, ranging from asymptomatic to severe presentations, even if core symptoms commonly include mild intellectual disability (ID) or sometimes learning difficulties, and sometimes speech impairment or language delay [12–14].

Despite advances in understanding CTD and the crucial role of Creatine in energy metabolism, the pathophysiological basis of the complex cognitive and behavioral impairment remains unclear. CTD is the most common cause of CCDS and is one of the leading causes of X-linked intellectual disability in males [15], representing a significant burden on healthcare systems and severely impacting both the patients’ and caregivers’ quality of life. Although the clinical spectrum of CTD has considerably expanded since the initial descriptions [16], and more than 20 pathogenic or likely pathogenic variants have been identified, no clear genotype-phenotype correlation has been established yet [17, 18] and no standardized treatment exists. While Creatine synthesis defects, such as AGAT and GAMT deficiencies, respond well to early oral Creatine supplementation [19, 20], CTD does not fully respond to any specific oral supplementation [15, 21, 22]. Literature still provides conflicting evidence regarding the benefits of Creatine and/or its precursors supplementation, such as Arginine and Glycine, in CTD. Although some of these supplementation therapies seem promising, the results remain insufficient to unequivocally establish long-term efficacy [23, 24]. Other recent studies, although needing further validation in clinical trials, propose innovative therapies, such as lipophilic Creatine analogs and betaine supplementation, in order to overcome lack of the cerebral Creatine Transporter [15, 22, 25].

Furthermore, the rarity of the disorder poses significant challenges for conducting systematic and rigorous comparative studies; consequently, the evidence supporting a standard treatment remains inconclusive.

Natural history studies and disease registries are critical for understanding how CTD evolves and for identifying potential subgroups of patients who may benefit from specific therapeutic approaches. There is an urgent need for reliable biomarkers with a translational value to assess therapeutic responses and monitor disease progression. Although some preclinical studies on animal models have shown promising results in defining quantitative and reliable biomarkers, these findings are still largely confined to the research domain and need further validations in humans [26].

We studied ten CTD patients and in this paper describe their long-term follow-up, ranging from 3 to 13 years after the initiation of therapy. Five of these patients, previously described [24], have continued their follow-up over the past decade, while the remaining five were diagnosed more recently and were followed for 3 to 5 years.

The primary aim of this study was to conduct a clinical follow-up of all the patients who had been referred to the IRCCS Stella Maris Foundation, Calambrone, Italy following the protocol we had previously established [24] and to provide insights into the therapeutic use of Creatine precursors. We also expanded the neuropsychological protocol to include two additional behavioural characteristics of CTD which had not been previously examined i.e.stereotypies and hyperactivity. A secondary objective was to monitor seizure activity in CTD patients undergoing oral supplementation therapy, so as to evaluate the potential additive effect of Arginine when used alongside conventional antiepileptic drugs in this population [22, 27].

## Materials and methods

### Patients

We began by collecting medical data through an eSurvey distributed via email to the main Italian centers which specialize in rare neurometabolic and neurogenetic diseases, in order to identify patients with a diagnosis of CTD. The online structured questionnaire was developed using Google Forms, and included information on gender, primary center of care, *SLC6A8* pathogenic or likely pathogenic variants type, and age at diagnosis for each participant. The questionnaire was filled in by the referring physician.

Subsequently, we conducted an in-person clinical assessment on the ten patients (P1-P10), belonging to eSurvey’s cohort, who referred to the IRCCS Stella Maris Foundation, Calambrone, Italy; these patients underwent regular long-term follow-up visits ranging from 3 to 13 years, after the introduction of oral Arginine supplementation. For five of these individuals (P1-P5) their early natural history, functional and imaging profile and a 36 month follow-up observation had been previously described [24]. We have continued following these patients over the past ten years and have added five additional patients who were diagnosed in the last decade and studied with the same follow-up protocol.

The study design for the CTD study outcome was approved by the Pediatric Ethics Committee of the Tuscany Region (CEPR code 201/2019). Informed consent was obtained from the parents or legal guardians.

### Follow-up measures: clinical assessment and investigations

Given the demographic heterogeneity and severity of the phenotype, we used a simplified protocol since a quantitative evaluation of functional skills would not have been possible with our previous approach [24]. All the patients in this observational study were subjected to the Adaptive Quotient (AQ) evaluation, according to the Vineland Adaptive Behavior Scales (VABS) or the Vineland Adaptive Behavior Scales second edition (VABS-II) [28], which have been suggested as valid outcome measures in CTD [29].

Their language receptive and expressive skills in terms of Equivalent Age (EA) [24] were also assessed, and an evaluation of the course of stereotypes and hyperactivity according to the Clinical Global Impression (CGI) Scale [30] was carried out. The CGI Scale was selected as an outcome measure given its established utility in both clinical practice and research [30, 31]. To date, the CGI has been widely used across diverse clinical domains and is recognized in regulatory contexts for its practicality and sensitivity as a clinician-rated global endpoint [32–34].

We analysed the above parameters at specific timepoints, to ensure data comparability across patients. T0 refers to the baseline visit and the start of treatment with Arginine; T1 and T2 visits after 12- and 36-months follow-up and treatment respectively; T3 visit refers to the last follow-up. We chose these observation times as they seemed to correspond for most patients, thus making the data comparable.

In the following paragraphs T3 only is extensively described as the protocol parameters regarding T0, T1 and T2 are already available for those described in 2012.

An extensive neuropsychological monitoring was possible in some patients only, where standardized tests such as Griffiths Scales and Performance Scales of Wechsler Preschool and Primary Scale of Intelligence (WPPSI) in preschoolers [35–39], Wechsler Intelligence Scale for children (WISC), Wechsler Adult Intelligence Scale IV Edition (WAIS-IV) in older subjects [40, 41], Leiter Scale 3rd edition [42] were used.

Clinical and ancillary investigations consisted of monitoring: (i) auxological data (weight, height, and OFC); (ii) routine laboratory evaluations (glycemia, BUN, creatininemia, hepatic enzymes, and urine analysis); (iii) Creatine/Creatinine (Cr/Crn) in urine; (iv) blood amino acids; (v) brain magnetic resonance proton spectroscopy (^1^H-MRS). (vi) Electroencephalogram (EEG) while awake and asleep; (vii) Electrocardiogram (ECG).

During follow-up patients were monitored annually by clinical and MRI/^1^H-MRS assays for the first 4 years after diagnosis, and subsequently by clinical evaluations only.

### Treatment protocol

After a CTD diagnosis, all the patients started oral supplementation therapy with Arginine 300 mg/kg/day alone or a combination of Arginine plus Creatine (200 mg/kg/day) and/or Glycine; at the outset of their enrollment in the follow-up program (T0), all the patients underwent monotherapy with Arginine, in accordance with the protocol previously delineated [24, 43].

## Results

Having completed our eSurvey, we identified 18 males who had been diagnosed with CTD in Italy, whose demographic and main clinical data are summarized in Table 1. Regular follow-up visits enabled us to select our cohort including the 10 patients who referred to IRCCS Stella Maris Foundation. Details of their main clinical, instrumental and laboratory data at diagnosis, along with the pharmacological and supplementation therapy at their more recent follow-up can be seen in Table 2. The clinical profiles of five newly diagnosed CTD patients included in the cohort, are extensively described below.

**Table 1:** eSurvey about CTD Italian males subjects: clinical/demographic data and mutational analysis

Subject	Gender (M/F)	Place of main medical care	SLC6A8 pathogenic or likely pathogenic variants	Age at diagnosis (y.m)
P1 [44]	M	IRCCS Stella Maris Foundation, Pisa	c.1006_1008del p.Asn336del De novo (pathogenic, known)	8.6
P2 [45]	M	IRCCS Stella Maris Foundation, Pisa	c.757 G > C p.Gly253Arg Maternally inherited (likely pathogenic, novel)	5.5
P3 [46]	M	IRCCS Stella Maris Foundation, Pisa	c.263–2 A > G Maternally inherited (pathogenic, novel)	2
P4 [47]	M	IRCCS Stella Maris Foundation, Pisa	c.1631 C > T p.Pro544Leu Maternally inherited (pathogenic, known)	5
P5 [24]	M	IRCCS Stella Maris Foundation, Pisa	c.1006_1008del p.Asn336del Maternally inherited (pathogenic, known)	17
P6*	M	IRCCS Stella Maris Foundation, Pisa	c.1255-24_1255-14del De novo (likely pathogenic, novel)	5.6
P7*	M	IRCCS Stella Maris Foundation, Pisa	c.1376T > C p.Leu459Pro (likely pathogenic, novel)	6.3
P8*	M	IRCCS Stella Maris Foundation, Pisa	c.757G > C p.Gly253Arg Maternally inherited (likely pathogenic, previously known in P2)	10.4
P9*	M	IRCCS Stella Maris Foundation, Pisa	c.1271_1291dup p.Gly424_Leu430dup Maternally inherited (likely pathogenic, novel)	4
P10*	M	IRCCS Stella Maris Foundation, Pisa	c.736_746del p.Val246Cysfs*47 (likely pathogenic, novel)	9
P11	M	IRCCS Foundation “Carlo Besta” Neurological Institute, Milan	c.1221_1223del p.Phe408del Maternally inherited (pathogenic, known)	1.6
P12	M	IRCCS Meyer, Florence	c.1304_1328del p.Ala435GlyfsTer20 (likely pathogenic, novel)	31
P13	M	IRCCS Meyer, Florence	c.1392+24_1393-30del (intronic region) De novo (pathogenic, known)	1.6
P14	M	University of Turin	c.1428 C > G p.Tyr476Ter De novo (pathogenic, novel)	18
P15	M	IRCCS Meyer, Florence	c.1169 C > T p.Pro390Leu Maternally inherited (pathogenic, known)	11
P16	M	Sapienza University, Rome	c.395–9 C > A Maternally inherited (likely pathogenic, novel)	7.10
P17°	M	IRCCS Giannina Gaslini, Genoa	c.803 C > G p.Pro268Arg Maternally inherited (likely pathogenic, novel)	9
P18°	M	IRCCS Giannina Gaslini, Genoa	c.803 C > G p.Pro268Arg Maternally inherited (likely pathogenic, novel)	32

°brothers. Data regarding the inheritance or de novo occurrence of the SLC6A8 pathogenic or likely pathogenic variants for P7 and P10 is not available: P7 was born from double heterologous invitro fertilization, while P10’s mother has never been traceable for segregation analysis. Abbreviations: M = Males; F = Females; IRCCS = Istituto di Ricovero e Cura a Carattere Scientifico; y.m = years.months. SLC6A8 pathogenic or likely pathogenic variants are reported according to international nomenclature standards

**Table 2:** Clinical, biochemical and imaging data in subjects of our cohort with long term follow up

Subject	CR/CRN urine	CR and GAA (P) (umOL/L)	MRI	Cognitive dysfunction	Seizures (Y/N)	Language	Behavior	Therapy at last follow-up visit
		**CR (U) (umol/L)**	**MRS-Brain CR**		**Age at onset (y.m)**			
P1 [44]	1.96	n.a.	Thin corpus callosum	Moderate (Leiter)	Y	Severe deficit Oral-motor dispraxia	Irritability	Arginine
		12543.66	Absent		1.0			
P2 [45]	3.08	CR 43.55 GAA n.a.	Normal	Mild (WPPSI-III)	N (only 2 isolated episodes of febrile convulsions in early childhood)	Severe deficit more impairment of verbal than nonverbal skills, oral-motor dispraxia	Anxiety, motor stereotypies	Arginine
		17184.00	Reduced					
P3 [46]	3.6	CR and GAA n.a.	Normal	Severe	Y	Severe deficit	Hyperactivity, irritability	Arginine + Risperidone + valproate
		n.a.	Reduced		2.0			
P4 [47]	1.83	n.a.	Normal	Moderate (Griffiths)	Y	Severe deficit	Hyperactivity, impulsiveness	Arginine + valproate (indication for gradual tapering until complete cessation)
		n.a.	Reduced		4.9			
P5 [24]	2.98	n.a.	White matter hyperintensity in the posterior regions	Severe	Y	Severe deficit	Aggressiveness, attention deficit, hyperkinetic behavior	Arginine + valproate + phenobarbital + levomepromazine + clonazepam
		n.a.	Reduced		8.0			
P6	2.24	CR 77.50 GAA 0.71	Normal	Mild (WISC-IV nonverbal subscales)	N	Mild deficit	ADHD	Arginine
		11066.83	Reduced					
P7	2.33	n.a.	Thin corpus callosum	Moderate (Griffiths)	Y	Severe deficit	Impulsiveness, oppositive-defiant	Arginine + valproate + clobazam + Risperidone
		23307.00	Reduced		1.2			
P8	2.54	n.a.	n.a.	Moderate (Leiter-3)	N	Absent	Aggressiveness, autistic-like behaviours, motor stereotypies	Arginine
		11452.04	Absent					
P9	4.99	n.a.	White matter hyperintensity in the posterior regions; thin corpus callosum	Moderate	Y	Absent	Autistic-like behaviours, hyperactivity, attention deficit	Arginine + carbamazepine + clobazam + Risperidone
		n.a.	Reduced		2.5			
P10	2.12	n.a.	Slight hyperintensity	Moderate (Leiter-3)	N	Severe deficit	ADHD	No therapy
		14474.00	Absent					

All data reported in the table refer to the time of CTD diagnosis, except for the last column, which shows the ongoing pharmacological/supplementation therapy at the last follow-up visit. The level of global developmental delay and cognitive dysfunction has sometimes been deduced from qualitative evaluations, in cases where patients were not amenable to standardized assessments. Conversely, when standardized tests were employed, this is specified in parentheses in the corresponding column

Abbreviations: CR = Creatine; CRN = Creatinine; P = Plasma; U = Urine; GAA = Guanidinoacetic Acid; n.a. = not available; MRS = Magnetic Resonance Spectroscopy; MRI = Magnetic Resonance Imaging; Y/N = Yes/No; y.m = years.months; WPPSI-III = Wechsler Preschool and Primary Scale of Intelligence 3^rd^ edition; WISC-IV = Wechsler Intelligence Scale for children 4^th^ edition; ADHD = Attention Deficit/Hyperactivity Disorder

Normal values: CR/CRN urine: < 1; CR (P): 18-141 umol/L; GAA (P): 0.22-3.14 umol/L; CR (U): 200-5500 umol/L

Due to the severe clinical presentation and the patients’ limited ability to cooperate only one attempt, which proved to be not always successful, was made in the majority of cases to quantitatively assess the level of global and cognitive development, mostly at CTD diagnosis. Therefore, when data from quantitative neuropsychological assessments were available, we documented them in the patient’s clinical history, albeit without incorporating them into our follow-up protocol. 

### Patient series

The five newly described patients with CTD were referred to the IRCCS Stella Maris, Pisa (P6, P7, P8, P10), and to the Meyer Children’s Hospital IRCSS, Florence (P9) due to intellectual disability, language disorder, behavioral issues and epilepsy. They underwent comprehensive metabolic work-ups (including blood and urine amino acids, Creatine, organic acids, and thyroid hormones levels) and genetic testing (array CGH and FMR1 analysis), following the diagnostic flowchart for cases of intellectual disability of unknown origin [48, 49]. MRI/^1^H-MRS exams further corroborated the suspicion of CCDS. The diagnosis was confirmed for 9 out of 10 patients by *SLC6A8* gene sequencing (Entrez Gene, GeneID: 6535) and quantitative analysis of Creatine and Creatinine in urine samples [25, 50, 51]. In four patients, a Creatine uptake assay was also performed. In one patient (P9), following unrevealing first-tier genetic testing, whole-exome sequencing (WES) was conducted, identifying a *SLC6A8* gene likely pathogenic variant. Diagnostic confirmation was subsequently achieved through a Creatine-specific metabolic assay on urine samples. The mean age at diagnosis in this group was 7.3 years (20 months − 17 years).

The parents of one patient (P10) declined the targeted therapy with Arginine, so we did not consider him further in the follow-up cohort, but described his clinical course as an example of natural history.

In patients with severe epilepsy, antiseizure medications (valproate, phenobarbital, carbamazepine) were added to the Arginine therapy.

**P1-P5**: The history and detailed clinical findings of these patients have already been reported [24, 44–47]. Their main characteristics are briefly summarized in Table 2.

**P6**: History of neuropsychiatric disorders on maternal side is reported (mother’s sister with mood disorders and severe drug addiction, one cousin deaf-mute). He was born at term with normal auxological parameters but showed early signs of global developmental delay and cognitive dysfunction and language impairments; he was initially diagnosed with behavioral problems, ADHD, mild language deficit, and mild intellectual disability. When 5.6 years old, the child underwent a diagnostic protocol that confirmed CTD. At physical examination, the head circumference was 50 cm (25-50th pc), stature was 121 cm (97th pc), and weight was 24 Kg (90th pc). Routine blood analyses were normal, including Creatine metabolites, in contrast with altered urine Creatine and abnormal Creatine/Creatinine ratio (see Table 2). Direct sequencing of SLC6A8 gene showed a de novo deletion in the intronic region c.1255-14_1255-24del, likely pathogenic.

Supplementation with Arginine (300 mg/Kg/day) and treatment with risperidone, determined a slight improvement in cerebral Creatine levels on ^1^H-MRS and in hyperactivity and language. According to the CGI score, a reduction in hyperactivity from 4.0 to 3.0 was observed. An improvement in expressive and receptive skills was noted, with an equivalent age that increased from 30 months to approximately 48 months. (For details see Additional file 1, Tables 3 and 4, and 5). Risperidone was later switched to Methylphenidate (MPH) and at last follow-up (4 years after starting Arginine), significant language impairment and behavioral issues persisted, with severe cognitive impairment (WISC-IV nonverbal subscale, VPI = 41). At the VMI test similar scores were obtained (SS = 47). Instability and impulsivity were still noted, even if a partial reduction was observed on MPH.

**P7**: Born via cesarean section after invitro fertilization, the patient exhibited mild global developmental delay and cognitive dysfunction during his first year. He experienced a first seizure at 16 months, followed by recurrent seizures that were poorly controlled by phenobarbital and later valproate. The first EEG, performed at another hospital after the initial seizure, showed mild fronto-central abnormalities, not further specified, while an MRI revealed a thin corpus callosum without significant alterations of myelination. At the age of 5, he was admitted to IRCCS Stella Maris Foundation due to early-onset epilepsy, global developmental delay and cognitive dysfunction, and thin corpus callosum. The head circumference was 52.5 cm (50-98th pc), stature was 113 cm (25th pc), and weight was 17.8 Kg (5th pc). The diagnostic work-up was performed: both the ECG and heart ultrasound were normal. Plasma aminoacids were normal, while a generalized hyperaminoaciduria with high levels of Glycine (2088 umol/mmol) was found in his urine; urinary organic acids were normal. Abnormal levels of Creatine in urine and altered Creatine/Creatinine ratio led to a suspicion of CTD (see Table 2). The NGS panel analysis for epilepsy-related genes revealed a hemizygous missense likely pathogenic variant (c.1376T > C p.Leu459Pro) in the *SLC6A8* gene. Impaired uptake of Creatine in fibroblasts was demonstrated. Arginine supplementation and valproate were prescribed, leading to improvement in motor skills, in emotional-behavioral control, and seizure control. However, seizures recurred after 5 years, hence necessitating the addition of clobazam. At the last follow-up (6 years after starting Arginine), severe language deficits, behavioral issues, and moderate ID were still present.

**P8**: This patient, with a non-contributory perinatal history and early normal development, experienced psychomotor regression when 3 years old, accompanied by severe behavioral problems. He was born in a rural area in China, and lived with his grandparents from the age of 5 months to 9 years, probably in a context of affective and environmental deprivation. Due to severe behavioural problems, he attended school only after his arrival in Italy. At age 10, the head circumference was 54 cm (50th pc), stature was 136 cm (10-25th pc), and weight was 29 Kg (10-25th pc). A metabolic assay revealed altered level of Creatine in urine sample and abnormal Creatine/Crn ratio (see Table 2). Direct sequencing of SLC6A8 gene showed a point likely pathogenic variant c.757G > C p.Gly253Arg, maternally inherited. Expressive language was almost absent and comprehension markedly impaired. Autistic-like traits, such as stereotypes, repetitive behaviors and an atypical sensory profile were also observed. The Leiter-3 cognitive test showed nonverbal abilities in the range of moderate intellectual disability (IQ value of 42). His adaptive profile, evaluated by the VABS II, was severely deficient for age (QA 20). Despite starting Arginine treatment, the patient showed minimal progression in abilities during the 3-year follow-up period, with persistent severe language impairment and autistic-like traits.

**P9**: Referred at age 4 for intellectual disability and epilepsy, this patient had a complicated pregnancy history that required isoxsuprine and absolute rest for his mother. He showed global developmental delay and cognitive dysfunction, pharmacoresistant frequent seizures and severe behavioral disturbances; at age 6 head circumference was 54 cm (50-75th pc), height 125 cm (75th pc), and weight 40 Kg (> 75th pc). An exome sequencing analysis on the family trio was carried out and revealed a maternally inherited 1271_1291dup p.Gly424_Leu430dup likely pathogenic variant in SLC6A8, maternally inherited. Arginine supplementation led to some improvement in attention, compliance in structured functional assessment and in seizure control, even if the clinical picture remained consistent with moderate/severe ID at the last follow-up. The child underwent two MRI/1H-MRS scans; however, the cerebral Creatine peak data are not comparable because the first scan was conducted on a different scanner. Language was absent, and hyperactivity and autistic-like traits markedly impaired the adaptive skills of the child.

**P10**: The first child of a Romanian mother with familial language impairment and a healthy Italian father was referred at age 9 for global developmental delay and cognitive dysfunction and behavioral problems. He was born at term by caesarean section and his earliest development milestones were appropriate. However, subsequent progress ceased, leading to a first assessment for global developmental delay and cognitive dysfunction at 14 months. Routine blood analyses showed microcytosis, hypochromia, and low creatinine levels (0.14 mg/dl; n.v. 0.67–1.17). An ECG revealed sinus tachycardia with marked arrhythmia, ST and anterior wave T anomalies while both an EEG and brain MRI were unrevealing. A diagnostic work-up revealed severe language deficits, leading to a diagnosis of moderate ID.

He was admitted to the IRCCS Stella Maris Foundation hospital at 9 years of age, for global developmental delay and cognitive dysfunction and behavioral problems. Routine blood analyses were normal; the ECG showed a long QT while the EEG showed normal background activity, with a photo-paroxysmal response between 15 and 30 Hz. During sleep, sharp spike-wave discharges appeared in the fronto-centro-temporal areas. Direct sequencing of SLC6A8 gene found a frameshift likely pathogenic (c.736_746del p.Val246Cysfs*47) causing premature protein truncation. The Creatine uptake was not measured due to the unavailability of fibroblasts.

Nonverbal cognitive testing (Leiter 3) revealed moderate Intellectual Disability. The patient presented severe language impairment producing syllabic sounds and vocalizations. He furthermore exhibited echolalia, in addition to severe hyperactivity and attention deficit. Arginine supplementation was discontinued after 6 months due to worsening behavioral symptoms. At his last visit, his expressive language was persistently characterized by severe dyspraxia with the production of single unintelligible words and his receptive language abilities seemed to be more preserved.

The qualitative cognitive evaluation which was conducted indicated severe ID with marked impairment in his daily-life adaptive skills also. Routine blood analyses were normal, except for creatinine (0.27 mg/dL, n.v. 0.40–0.73), ferritin (15 ug/L, nv 30–400), and INR (1.15, n.v. 0.87–1.12). The auxological parameters remained stable: body weight 50-75th C, height 75th C, head circumference 25-50th C. The Creatine in the urine sample was 9671 umol/L and the ratio Creatine/Creatinine 2.48.

### Longitudinal follow-up and outcomes

In the five previously described patients [24], the young adults’ global clinical-functional profile remained characterized by moderate to severe ID, language deficits, with a frequent component of dyspraxia, some with epilepsy, and clinical-laboratory findings confirming the characteristic metabolic alterations of Creatine. After an initial and encouraging period of stability or slight improvement during the first 36 months of Arginine supplementation [24], the functional profile worsened particularly regarding adaptive skills, motor hyperactivity and stereotyped behavior.

**Fig. 1:**
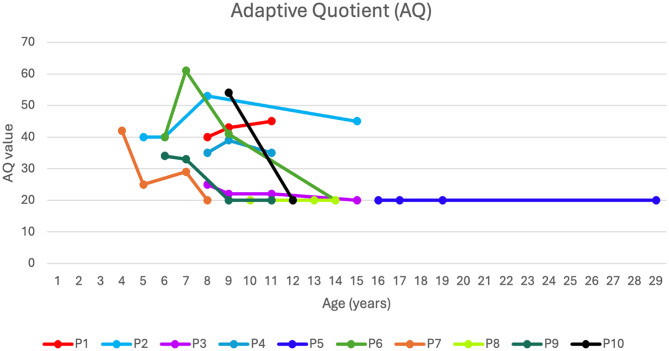
The trajectory of the adaptive behavior composite score across the designated follow-up time points. The graph illustrates the trend of the Adaptive Quotient composite score (AQ) in individual patients of our cohort at the available timepoints (T0, T1, T2, and T3). The y-axis represents the AQ value, the x-axis indicates age in years. The available AQ scores for P10, the patient who discontinued treatment with Arginine, are also shown

When looking at the overall trend of the clinical features, the adaptive skills, which were assessed with the VABS or VABS II that had been answered by the parent/caregiver, the mean adaptive quotient was 34 at T0, corresponding to a moderate-severe level of deficit, although in some subjects slightly higher adaptive quotients were observed (see Additional file 1, Table 1). At T1, a substantial stability in the adaptive quotient was observed, along with a slight improvement in scores among some patients (P1, P4, P6) (Fig. 1). On the contrary, P9, one of the epilepsy patients in our cohort, despite beginning Arginine treatment earlier than the other subjects, experienced a significant decline in adaptive functioning as early as 12 months after starting the supplementation therapy. At T2, the trend in adaptive quotient appeared more variable across different patients, with a slight overall decline (see Additional file 1, Table 1), although some patients still exhibited a mild improvement (P1, P2) (Fig. 1). Overall, patients with poorer adaptive skills from T0, due to the severity of their general clinical picture, maintained a nearly stable trend over in adaptive functioning (P3, P5, P8) (Fig. 1). The patient without Arginine treatment dropped out of follow-up (P10) and experienced a more rapid decline in his adaptive skills.

By dividing our cohort into epileptic and non-epileptic subjects and qualitatively analyzing the mean adaptive quotient in the two groups, a gradual but consistent decrease was observed from T1 onwards in the epileptic group. In contrast, the non-epileptic group showed a slight increase in the quotient at T1, followed by a subsequent decrease, while remaining at higher quotient levels compared to the epileptic patients (see Additional file 1, Table 1). 

The graph illustrates the trend of the Adaptive Quotient composite score (AQ) in individual patients of our cohort at the available timepoints (T0, T1, T2, and T3). The y-axis represents the AQ value, the x-axis indicates age in years. The available AQ scores for P10, the patient who discontinued treatment with Arginine, are also shown.

**Fig. 2:**
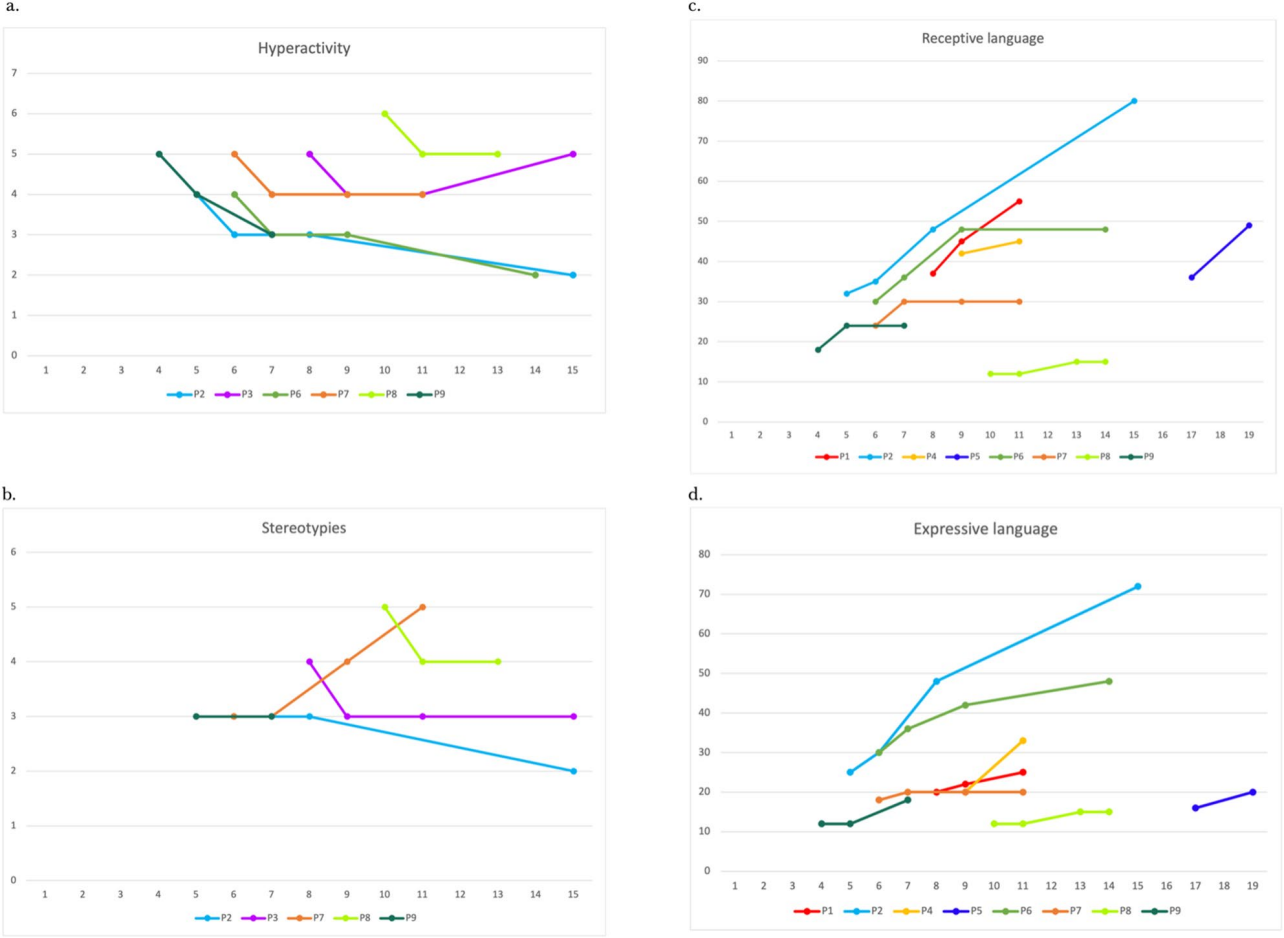
Hyperactivity, stereotypies and language skills’ trend across the designated follow-up time points. All data refer to patients from the cohort who underwent longitudinal assessment at the established time points (T0, T1, T2, T3) for hyperactivity (**a**), Stereotypies (**b**), and language skills (**c**, **d**). **a**, **b**. The y-axis indicates the CGI values, while the x-axis indicates age in years. **c**. The y-axis represents the equivalent age (EA) in months for receptive language abilities, the x-axis indicates the chronological age in years. **d**. The y-axis represents the EA in months for expressive language abilities, the x-axis indicates the chronological age in years

Hyperactivity and stereotypies, which had not been assessed in the previous study, but were measured here according to the CGI criteria, were reduced almost uniformly after Arginine was started, both in those patients who had already been described [24] and in the newly diagnosed individuals; specifically, regarding hyperactivity, the available data (P2, P3, P6, P7, P8, and P9) indicate a uniform one-point reduction on the CGI score at T1 compared to T0, with a trend of substantial stability observed at both T2 and T3. P3 experienced a new increase in hyperactivity at T3 (Fig. 2a).

Similarly, in terms of stereotypies, the available data (P2, P3, P7, P8, and P9) show a general stability in CGI scores at both T1 and T2 (with P3 and P8 showing a one-point CGI reduction at T1). P7 demonstrated a worsening trend after 36 months of stability, with a two-point CGI increase at T3 compared to T0 (Fig. 2b).

Analyzing the two groups of epileptic and non-epileptic subjects (see Additional file 1, Tables 2 and 3), in terms of stereotypies, although many data were unavailable (mean CGI at T0 and T3 in non-epileptics, due to data being available for only one patient), a substantial stability in the non-epileptic group was observed, whereas the epileptic group showed a temporary slight improvement between T0 and T1, followed by a subsequent increase in symptom severity according to the CGI score. Regarding hyperactivity, there seems to be an improvement in symptoms up to 36 months after the start of Arginine treatment in both groups. After 36 months, while the non-epileptic group continued to show a decrease in CGI scores, the epileptic group experienced a new increase in scores reflecting symptom severity.

As far as language abilities, which were examined by defining an Equivalent Age (EA) level, are concerned, a global trend of slight improvement or substantial stability was observed until the last follow-up, in both verbal comprehension and production, although linguistic abilities remain significantly below the expected level for the chronological age (see Additional file 1, Tables 4 and 5). In particular: (a) Receptive language: at T1, a slight and consistent improvement was observed across all patients for whom language assessments were possible; at T2, a further slight improvement (P1, P2, P4, P5, P6) or stability in receptive abilities (P7, P9) was detected. Given the severity of the initial overall clinical condition, P8 remained nearly stable, with an EA of 12–15 months in receptive language abilities (Fig. 2c); (b) Expressive language: at T1, a slight improvement (P2, P6) or stability (P1, P7, P9) was observed compared to T0; similarly, at T2, a further improvement (P2, P4, P5, P6, P9) or stability (P1, P7) was detected compared to previous timepoints. Due to the severity of the initial overall clinical condition, P8 remained nearly stable at an equivalent age of 12–15 months in expressive abilities also (Fig. 2d).

No significant differences in terms of language improvement were observed between epileptic and non-epileptic group (see Additional file 1, Tables 4 and 5).

The data acquired at the first ^1^H-MRS highlighted that all the patients in this cohort exhibited a markedly reduced cerebral Creatine peak, which was sometimes below the detection limit. Subsequent assessments after 12–18 months of Arginine treatment demonstrated a slight increase in Creatine concentration, after which cerebral Creatine remained constant over time in all patients (P1-P9). P10 dropped out of the follow-up protocol, and so no further MRI/^1^H-MRS evaluations were possible.

With the exception of patient P6, ¹H-MRS follow-up examinations in the remaining cases were performed on different scanners over time, which precludes a reliable quantitative comparison of the resulting data across patients.

**Fig. 3:**
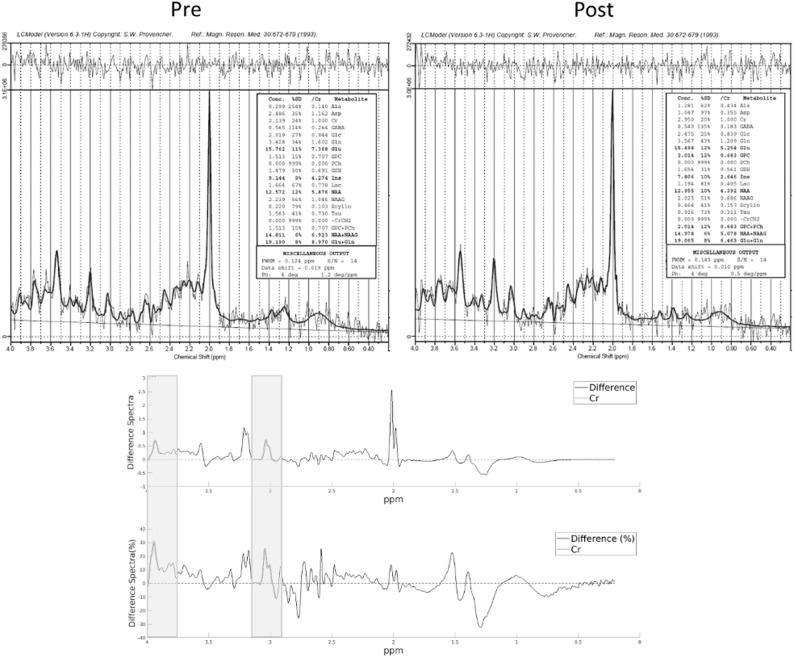
^1^H-MRS data pre- and post- Arginine supplementation in P6. **Top**: ^1^H spectra of S1 pre- (left) and post-treatment (right) after baseline subtraction, as estimated by Lcmodel. Upper panel shows the fit resduals. **Bottom**: Difference between the spectral fit estimated by LCModel (Pre-Post). Top panel shows the absolute difference; bottom panel shows the percentual change. The two Creatine peaks are highlighted. The positive absolute difference and the relative difference show an increment in cerebral Creatine concentration of 27.5%, higher than variations that can be attributed to noise

In Fig. 3 we report the ^1^H-MRS spectra acquired before and after 18 months of treatment in P6: the spectra were analyzed with LCModel, the absolute and the relative difference demonstrated an increment in cerebral Creatine concentration of approximately 25–30%.

In our cohort, 6 patients out of 10 were diagnosed with epilepsy. All of them began supplementation treatment with Arginine. By monitoring the epileptic clinical course at T1, T2, and at the last follow-up performed (T3), it is possible to detect a substantial stability of seizure burden (P7) or a progressive reduction in the frequency of seizures (P1, P3, P4, P5, P9). One patient (P5), on the 13th year of follow-up, began tapering off valproic acid and then completely discontinued it, due to seizure freedom for seven years. After Arginine supplementation was started, P4 was switched from antiseizure medications polytherapy – which had provided only partial seizure control– to monotherapy with valproic acid, achieving excellent control. Another patient in our cohort (P1), following 12 months of Arginine monotherapy, exhibited no further epileptic seizures. Consequently, two years later, the antiseizure regimen was gradually tapered off. In subsequent contacts with referring physician parents reported the cessation of Arginine intake which led to the re-emergence of epileptic seizures, necessitating the reintroduction of antiseizure medications (topiramate + carbamazepine). Furthermore, an exacerbation of psychiatric symptoms characterized by psychotic episodes with severe emotional-behavioral dysregulation occurred, prompting the initiation of pharmacotherapy with risperidone. For specifics regarding the epileptological clinical course in patients in our cohort, see Table 3.

**Table 3:** Clinical trend of seizures in epileptic patients of our cohort at designated timepoints

Subject	T0 Seizure type	T1 Seizure type	T2 Seizure type	T3 Seizure type
	**Seizure frequency**	**Seizure frequency**	**Seizure frequency**	**Seizure frequency**
P1	Focal seizures with occipital onset	No seizures	No seziures	No seizures
	Sporadic (2–3 per year)			
P3	Myoclonic and Asymmetrical spasms	Myoclonic and Asymmetrical spasms	No seizures	Almost no seizures (a single episode in 3 years)
	every day in a week	some days in a week		
P4	Previous history of severe epilepsy with status epilepticus and polymorphic seizures (generalized, focal, myoclonic), followed by sporadic generalized tonic-clonic seizures	Seizures controlled only by monotherapy with valproate	Seizures controlled only by monotherapy with valproate	Seizures controlled only by monotherapy with valproate
	n.a. (partially controlled by multidrug therapy with ASMs)			
P5	Secondary Generalized Focal at onset	No seizures	Almost no seizures (a single episode in 2 years)	No seizures
	Sporadic (3–4 per year)			
P7	Focal, sometimes with secondary generalization	Focal seizures, sometimes with secondary generalization	Almost no seizures (a single episode in 2 years)	Focal, sometimes with secondary generalization
	Sporadic (1–2 per year)	Sporadic (1–2 per year)		Sporadic (2–3 per year)
P9	Polymorphic (generalized and focal)	No seizures	Focal seizures	Only one cluster of 3 seizures in hyperpyrexia in the last year
	Every day in a week		Every day in a week	

T0 refers to the start of the treatment with Arginine; T1 refers to 12 months follow-up after T0; T2 refers to 36 months follow-up after T0; T3 refers to the last follow-up visit. Abbreviations: n.a. = not available; ASMs = Antiseizure Medications

### Summary of results after Arginine supplementation

Following the beginning of Arginine supplementation, notable changes were observed in seizure control, hyperactivity, and stereotyped behaviors among the nine subjects of our cohort.

Regarding seizure control, the clinical course for the six patients with epilepsy showed generally positive outcomes: five patients (P1, P3, P4, P5, and P9) experienced a progressive reduction in seizure frequency, while one patient (P7) showed a substantial stabilization of the seizure burden. These improvements were significant enough in some cases to warrant changes in medication: P5 achieved seizure freedom for seven years, allowing for the eventual discontinuation of valproic acid. Similarly, P4 transitioned from a partially effective polytherapy to a valproic acid monotherapy with excellent results. Patient P1 became seizure-free after 12 months of arginine monotherapy, leading to the withdrawal of antiseizure medication two years later; when he stopped the supplementation and experienced a re-emergence of epileptic seizures, which required the reintroduction of antiseizure drugs.

In terms of hyperactivity, an almost uniform reduction of symptoms in all patients for whom data was available (P2, P3, P6, P7, P8, and P9) was noted after the beggining of Arginine supplementation. This improvement remained largely stable for up to 36 months. However, after this period, the response diverged between the epileptic and non-epileptic groups. While the non-epileptic group continued to show improvement, the epileptic group experienced a new increase in the severity of hyperactivity symptoms. Patient P3 was an exception, showing a renewed increase in hyperactivity at a later follow-up.

The effects on stereotyped behaviors were more varied. Initially, a general stability was observed, with some patients (P3, P8) showing a one-point reduction on the CGI scale at the first follow-up. Over time, the non-epileptic group maintained a substantial stability. In contrast, the epileptic group showed a slight temporary improvement initially, which was followed by a subsequent increase in symptom severity.

Overall, after an initial period of stability or slight improvement, the long-term functional profile of patients tended to worsen, particularly regarding motor hyperactivity and stereotyped behavior.

## Discussion

CTD, although rare, is one of the most common causes of X-linked intellectual disability in males, second only to Fragile X syndrome [22, 52, 53]. The prevalence of CTD may be underestimated due to overlapping features with other syndromic conditions or neurodevelopmental disorders. Diagnostic delays are common, with the age of diagnosis ranging from 2 to 20 years, despite early clinical signs that could suggest CTD [54]. In our study cohort, the average age at diagnosis was 7.3 years, with a range from 1.6 to 17 years.

The CTD syndrome features a broad spectrum of clinical manifestations [1, 22]. The most common include early childhood onset global developmental delay and cognitive dysfunction. Epilepsy affects approximately 60% of individuals [17, 25, 55]. Autistic-like features and behavioral difficulties such as hyperactivity, attention deficit, impulsivity and aggressiveness, are also common. Other neurological symptoms such as hypotonia, spasticity, and movement disorders may also be present, along with non-neurological symptoms affecting the gastrointestinal and cardiovascular systems: in particular, regarding cardiac abnormalities, a prominent finding in male CTD patients is a prolonged QTc interval, associated with an increased sudden death risk, alongside frequent T-wave abnormalities like inversion and flattening [10, 56]; in line with this report, ECG abnormalities were found in one subject of our cohort (P10). Furthermore, echocardiographic analysis in CTD patients can reveal increased left ventricular internal dimension and ventricular wall thinning despite preserved systolic function, suggesting a developing dilated cardiomyopathy [56]. In our cohort, none of the patients showed echocardiographic abnormalities.

Dysmorphic features are occasionally observed [15, 25, 29]. Our cohort of 10 patients exhibited all the clinical characteristics most frequently associated with CTD.

A variety of pathogenic or likely pathogenic variants, affecting the *SLC6A8* gene, have been identified in CTD. Missense pathogenic or likely pathogenic variants and single amino acid deletions are the most frequent. While missense pathogenic or likely pathogenic variants are generally associated with a milder phenotypic presentation, exceptions exist [17, 57]. In our cohort, patients P1 and P5, who share the same SLC6A8 gene deletion, exhibited severe language impairment and moderate to severe intellectual disability (ID) at diagnosis. Patients P2 and P8, carrying the same missense likely pathogenic variant, manifested significant language impairment and mild to moderate ID, with a relatively benign epilepsy phenotype. However, due to the small sample size, it is not currently possible to identify a specific phenotypic profile for certain SLC6A8 variants, leaving genotype-phenotype correlations elusive, as also suggested in a larger cohort [29].

Since the first patient with CTD was described in 2001 [16], various supplementation strategies have been explored. A recent review [15] suggests that polytherapy with Creatine and its precursors or a combination of precursors alone led to improvements in epilepsy and behavioral aspects in 32.2% of treated patients. Emerging evidence indicates that epilepsy in CTD patients may be related to brain energy metabolism depletion and mitochondrial dysfunction, with Creatine itself showing potential anticonvulsant effects [15, 58]. In line with previous reports, our cohort showed stabilization or slight improvement in linguistic and adaptive skills during the first 12 months of Arginine supplementation. However, when Arginine therapy was discontinued, a significant deterioration in behavioral aspects was observed, including increased emotional and behavioral dysregulation, attention deficit, restlessness, and impulsivity. The improvements observed with Arginine supplementation do not seem to be long-lasting, particularly regarding aspects of behavior. At the 36-month follow-up, or the long follow-up from the start of oral supplementation, a decline or lack of improvement in adaptive skills was noted (see Additional file 1, Table 1). Hyperactivity and motor stereotypes were also monitored using the Clinical Global Impression (CGI) scale, showing initial slight improvements that later stabilized or worsened (see Additional file 1, Tables 4 and 5).

Given the clinical heterogeneity of patients in our cohort, often presenting with a severe clinical phenotype from T0, and considering the limited sample size, it was not possible to determine whether an early start of Arginine treatment correlates with improved long-term outcomes compared to a later initiation of supplementation. Therefore, further studies on this topic are needed, as they may support the importance of early diagnosis and treatment. In this regard, future multicenter collaborative studies would be essential to increase sample size, reduce variability, and allow more robust conclusions on the timing and efficacy of therapeutic interventions in CTD.

Quantitative comparison of the ^1^H-MRS spectra obtained at T0 and after 18 months of Arginine treatment in P6 showed a 25–30% increase in cerebral Cr, followed by subsequent stabilization over time. To the best of our knowledge, no similar increase has been reported in the literature, as existing data indicate a substantial stability of the spectroscopic profile even after the initiation of supplementation therapy, either in mono- or polytherapy [59, 60]. Therefore, we believe that our finding provides a basis to encourage future studies in this direction, expanding the sample size in order to confirm or refute the statistical significance of this observation.

At T0, patients exhibited variable seizure presentations in terms of frequency and type, despite ongoing antiseizure medication.

Following Arginine supplementation, a general reduction in seizure frequency was observed. Furthermore, in P1 seizures re-exacerbated when Arginine was discontinued. Since all patients continued treatment with at least one antiseizure medication after starting Arginine supplementation, these data suggest a potential synergistic role of Arginine with conventional antiseizure medications in reducing the clinical manifestation of epilepsy. Arginine alone could also aid in simplifying antiseizure therapy, by allowing a switch from polytherapy to monotherapy (P4).

A qualitative analysis of our cohort indicates that non-epileptic subjects tend to show slightly better outcomes than epileptic ones, particularly in adaptive functioning and hyperactivity, whereas the progression of language skills appears comparable between the two groups.

Another interesting finding in epilepsy patients in our cohort is the correlation between severity of baseline epileptic encephalopathy, which develops from the first years of life, and the severity of the clinical phenotype (stereotypies, hyperactivity, language disorder, adaptive skills). Epilepsy patients with a high recurrence of ictal events also show greater resistance to potential improvement in their functional profile following the beginning of Arginine supplementation (P3, P7).

Taken together, these data underscore the importance of an early diagnosis and the timely epilepsy management in CTD patients, to promote a better neurocognitive and behavioral outcome, although variable over time. In fact, considering the divergent responses in terms of adaptive and behavioral outcomes in the group of non-epileptic compared to epileptic subjects, we might speculate on an important role of altered cerebral electrical activity and uncontrolled seizure recurrence in determining a more severe phenotype and in worsening the response to arginine therapy.

While the stabilization of seizures appears to have longer-lasting effects, the clinical benefits of supplementation therapy on behavioral or neuropsychological aspects remain limited. Only about one-third of treated patients experienced significant improvement, with neuropsychological and behavioral profiles often deteriorating over time [21, 22].

Hyperhomocysteinemia has been reported in CTD patients under glycine and arginine treatment [61], and its evaluation may represent an important parameter for monitoring metabolic imbalance and treatment safety. In our study, however, plasma amino acid quantification was considered an ancillary investigation and was therefore not performed systematically in all patients, even if few dosage performed demonstrated normal values (P1-P5, P7).

The lack of data on disease history and quantitative biomarkers hampers the development of effective treatments for CTD [26]; neither ^1^H-MRS nor neuropsychological assessments seem effective for monitoring disease progression. The use of ^1^H-MRS has limited use, partly due to the need for sedation in most patients, and it does not reveal significant changes in brain Creatine levels over time [24, 59]. Neuropsychological assessments are also challenging due to phenotypic heterogeneity, which often makes quantitative assessment inapplicable. The Creatine/Crn ratio in urine is effective for CTD screening in males but is less useful as a biomarker for monitoring therapeutic strategies, as biochemical and ^1^H-MRS data remained stable over time despite clinical changes following supplementation therapy. Recent efforts in animal models have shown promise if EEG recordings and hemodynamic changes at the brain level, such as IOS recording and functional near-infrared spectroscopy (fNIRS) are considered, but further studies are needed for validation [25, 26].

## Conclusions

Our cohort of CTD subjects exhibited a spectrum of clinical manifestations consistent with those documented in previous literature. Despite the variability in clinical presentation, we identified certain common traits that emphasize the critical importance of early diagnosis and timely intervention. Treatment with Arginine supplementation yielded some promising outcomes, particularly in stabilizing or marginally improving linguistic and adaptive skills. However, the long-term efficacy of Arginine supplementation, especially when aspects of behavior are taken into consideration, remains uncertain. This uncertainty underscores the need for ongoing research into alternative therapeutic approaches to better address the multifaceted challenges posed by CTD. In addition to clinical observations, our study highlights the potential of emerging biomarkers, identified in animal models, to improve disease monitoring and therapeutic assessment in CTD patients. While these biomarkers show promise, further validation in human studies is crucial to translate these findings into effective clinical practice. Among the various modalities, EEG and functional near-infrared spectroscopy (fNIRS) stand out as the most promising tools for the longitudinal monitoring of disease progression and for evaluating the efficacy of new therapeutic interventions. The continued exploration of these techniques holds the potential to significantly improve outcomes for CTD patients and their families.

## Supplementary Information

Below is the link to the electronic supplementary material.

Supplementary Material 1

## Data Availability

The datasets supporting the conclusions of this article are included within the article and its Additional files.
