# Supplementary material for: Advancing clinical insight into creatine transporter deficiency: long term outcome and new observations from the Italian cohort
Source: Orphanet J Rare Dis. 2026 Mar 3;21:135. doi: 10.1186/s13023-026-04289-3 (PMC13063627; doi:10.1186/s13023-026-04289-3)
Supplement: Supplementary file 1 — Supplementary Material 1 [file 13023_2026_4289_MOESM1_ESM.docx]

**Table 1. Adaptive Behavior Composite score in our cohort of patients who underwent Arginine treatment**

| Epileptic subjects | T0 | T1 | T2 | T3 |
| --- | --- | --- | --- | --- |
| P1 [33] | 40 | 43 | 45 | n.a. |
| P3 [35] | 25 | 22 | 22 | 20 |
| P4 [36] | 35 | 39 | 35 | n.a. |
| P5 [13] | 20 | 20 | 20 | 20 |
| P7 | 34 | 33 | 20 | 20 |
| P9 | 42 | 25 | 29 | 20 |
| Mean ABC score  ± SD | 33  ± 9 | 30  ± 9 | 28  ± 10 | 20  ± 0 |
| Non epileptic subjects | **T0** | **T1** | **T2** | **T3** |
| P2 [34] | 40 | 40 | 53 | 45 |
| P6 | 50 | 61 | 41 | 20 |
| P8 | 20 | 20 | 20 | 20 |
| Mean ABC score  ± SD | 37  ± 15 | 40  ± 20 | 38  ± 17 | 32  ± 13 |
| Total mean ABC score  ± SD | 34  ± 10 | 34  ± 14 | 32  ± 12 | 24  ± 9 |

T0 refers to the start of the treatment with Arginine; T1 refers to 12 months follow-up after T0; T2 refers to 36 months follow-up after T0; T3 refers to the last follow-up visit.

Abbreviations: ABC = Adaptive Behavior Composite; SD = Standard Deviations; n.a. = not available

**Table 2. Stereotypies severity according to CGI scale in our cohort of patients who underwent Arginine treatment**

| Epileptic subjects | T0 | T1 | T2 | T3 |
| --- | --- | --- | --- | --- |
| P1 [33] | n.a. | n.a. | n.a. | n.a. |
| P3 [35] | 4.0 | 3.0 | 3.0 | 3.0 |
| P4 [36] | n.a. | n.a. | n.a. | n.a. |
| P5 [13] | n.a. | n.a. | n.a. | n.a. |
| P7 | 3.0 | 3.0 | 4.0 | 5.0 |
| P9 | n.a. | 3.0 | 3.0 | n.a. |
| Mean GCI score  ± SD | 3.5  ± 0.7 | 3.0  ± 0.0 | 3.3  ± 0.6 | 4.0  ± 1.4 |
| Non epileptic subjects | **T0** | **T1** | **T2** | **T3** |
| P2 [34] | n.a. | 3.0 | 3.0 | 2.0 |
| P6 | n.a. | n.a. | n.a. | n.a. |
| P8 | 5.0 | 4.0 | 4.0 | n.a. |
| Mean CGI score  ± SD | n.a | 3.5  ± 0.7 | 3.5  ± 0.7 | n.a. |
| Total mean CGI score  ± SD | 4.0  ± 1.0 | 3.2  ± 0.4 | 3.4  ± 0.5 | 3.3  ± 1.5 |

T0 refers to the start of the treatment with Arginine; T1 refers to 12 months follow-up after T0; T2 refers to 36 months follow-up after T0; T3 refers to the last follow-up visit. Abbreviations: n.a. = not available; CGI = Clinical Global Impression scale; SD = Standard Deviations

**Table 3. Hyperactivity severity according to CGI scale in our cohort of patients who underwent Arginine treatment**

| Epileptic subjects | T0 | T1 | T2 | T3 |
| --- | --- | --- | --- | --- |
| P1 [33] | n.a. | n.a. | n.a. | n.a. |
| P3 [35] | 5.0 | 4.0 | 4.0 | 5.0 |
| P4 [36] | n.a. | n.a. | n.a. | n.a. |
| P5 [13] | n.a. | n.a. | n.a. | n.a. |
| P7 | 5.0 | 4.0 | 4.0 | 4.0 |
| P9 | 5.0 | 4.0 | 3.0 | n.a. |
| Mean GCI score  ± SD | 5.0  ± 0.0 | 4.0  ± 0.0 | 3.7  ± 0.6 | 4.5  ± 0.7 |
| Non epileptic subjects | **T0** | **T1** | **T2** | **T3** |
| P2 [34] | 4.0 | 3.0 | 3.0 | 2.0 |
| P6 | 4.0 | 3.0 | 3.0 | 2.0 |
| P8 | 6.0 | 5.0 | 5.0 | n.a. |
| Mean CGI score  ± SD | 4.7  ± 1.1 | 3.7  ± 1.1 | 3.7  ± 1.1 | 2.0  ± 0.0 |
| Total mean CGI score  ± SD | 4.8  ± 0.7 | 3.8  ± 0.7 | 3.7  ± 0.8 | 3.2  ± 1.7 |

T0 refers to the start of the treatment with Arginine; T1 refers to 12 months follow-up after T0; T2 refers to 36 months follow-up after T0; T3 refers to the last follow-up visit. Abbreviations: n.a. = not available; CGI = Clinical Global Impression scale; SD = Standard Deviations

**Table 4. Receptive language trend in our cohort of patients who underwent Arginine treatment**

| Epileptic subject | T0 | T1 | T2 | T3 |
| --- | --- | --- | --- | --- |
| P1 [33] | 37.0 | 45.0 | 55.0 | n.a. |
| P3 [35] | n.a. | n.a. | n.a. | n.a. |
| P4 [36] | n.a. | 42.0 | 45.0 | n.a. |
| P5 [13] | n.a. | 36.0 | 49.0 | n.a. |
| P7 | 24.0 | 30.0 | 30.0 | 30.0 |
| P9 | 18.0 | 24.0 | 24.0 | n.a. |
| Mean EA  ± SD | 26.3  ± 9.7 | 35.4  ± 8.6 | 40.6  ± 13.1 | n.a. |
| Non epileptic subjects | **T0** | **T1** | **T2** | **T3** |
| P2 [34] | 32.0 | 35.0 | 48.0 | 80.0 |
| P6 | 30.0 | 36.0 | 48.0 | 48.0 |
| P8 | 12.0 | 12.0 | 15.0 | 15.0 |
| Mean EA  ± SD | 24.7  ± 11.0 | 27.7  ± 13.6 | 37.0  ± 19.0 | 41.0  ± 23.3 |
| Total mean EA  ± SD | 25.5  ± 9.3 | 32.5  ± 10.5 | 39.2  ± 14.3 | 43.2  ± 28.0 |

Receptive language level is expressed in terms of Equivalent Age (months) at different timepoints. T0 refers to the start of the treatment with Arginine; T1 refers to 12 months follow-up after T0; T2 refers to 36 months follow-up after T0; T3 refers to the last follow-up visit. Abbreviations: n.a. = not available; EA = Equivalent Age; SD = Standard Deviations

**Table 5. Expressive language trend in our cohort of patients who underwent Arginine treatment**

| Epileptic subjects | T0 | T1 | T2 | T3 |
| --- | --- | --- | --- | --- |
| P1 [33] | 20.0 | 22.0 | 25.0 | n.a. |
| P3 [35] | n.a. | n.a. | n.a. | n.a. |
| P4 [36] | n.a. | 20.0 | 33.0 | n.a. |
| P5 [13] | n.a. | 16.0 | 20.0 | n.a. |
| P7 | 18.0 | 20.0 | 20.0 | 20.0 |
| P9 | 12.0 | 12.0 | 18.0 | n.a. |
| Mean EA  ± SD | 20.8  ± 8.3 | 22.0  ± 8.0 | 29.2  ± 12.1 | n.a. |
| Non epileptic subjects | **T0** | **T1** | **T2** | **T3** |
| P2 [34] | 25.0 | 30.0 | 48.0 | 72.0 |
| P6 | 30.0 | 36.0 | 42.0 | 48.0 |
| P8 | 12.0 | 12.0 | 15.0 | 15.0 |
| Mean EA  ± SD | 22.3  ± 9.3 | 26.0  ± 12.5 | 35.0  ± 17.6 | 42.3  ± 25.0 |
| Total mean EA  ± SD | 19.5  ± 7.1 | 21.0  ± 8.4 | 27.6  ± 12.1 | 38.7  ± 26.5 |

Expressive language level is expressed in terms of Equivalent Age (months) at different timepoints. T0 refers to the start of the treatment with Arginine; T1 refers to 12 months follow-up after T0; T2 refers to 36 months follow-up after T0; T3 refers to the last follow-up visit. Abbreviations: n.a. = not available; EA = Equivalent Age; SD = Standard Deviations
